# PReS-FINAL-2365: Southern hemisphere educational partnership for pediatric arthritis and rheumatological diseases (Sheppard): pediatric rheumatology without borders

**DOI:** 10.1186/1546-0096-11-S2-O30

**Published:** 2013-12-05

**Authors:** RA Russo, MM Katsicas, K Webb, C Scott

**Affiliations:** 1 Immunology & Rheumatology, Hospital De Pediatría Garrahan, Buenos Aires, Argentina; 2 Rheumatology, Red Cross Memorial Children's Hospital, Cape Town, South Africa

## Introduction

Educational strategies and contents developed in the First World are not always adapted to the needs and challenges of developing countries. Cooperation and collaboration between developing countries can be mutually beneficial and create the basis for a longstanding, Third World-centered educational program aimed at increasing the pediatric support for Pediatric Rheumatology (PR).

## Objectives

To describe a binational (Argentina-South Africa), bicontinental, educational strategy aimed at increasing the pediatric support for PR.

## Methods

This is an ongoing educational, international, project that aims at combining the expertise and strategies from 2 centers in different developing countries with similar health problems and needs in order to improve the care of children with Rheumatic Diseases. This is a project supported by an ILAR educational grant. The objectives of the educational program are: 1) To provide pediatricians with education and training in the management of patients with juvenile rheumatic diseases in order to facilitate early diagnosis, referral and follow up. 2) To establish and develop permanent communication means for bidirectional contact between local teams and specialized centers. 3) To establish an integrated South Africa-Argentina (SA-A) teaching strategy, adapted to the needs of these developing countries. Strategies used were: selection of 6 pediatricians working in underserved areas of SA-A; 3 months-long rotations of in Pediatric Rheumatology centers in Cape Town and Buenos Aires; supervised patient-centered education and personalized training of pediatricians where they applied to a 10-item curriculum-based program; visiting professorships to those areas for the development of education of basics of PR for pediatricians through patient-based teaching sessions and interactive discussions; periodic, problem-based, SA-A teleconference rounds. A tailored educational program included specific competency-based educational goals for each trainee as well as community-based epidemiological research and team-based, patient-oriented care. Post-training assessment of trainees' skills and knowledge is carried through multiple choice tests and Objective Structured Clinical Examination, as well as trainees' satisfaction through a structured survey.

## Results

Six pediatricians (3 in Argentina and 3 in South Africa) received a comprehensive training according to the educational program. They were successfully evaluated and returned to their local setting, where they are currently involved in the care of children and adolescents with rheumatic conditions. Their activities are periodically monitored through combined trainer-trainee case discussions. Visiting professorships resulted in the delivery of basics of PR to over 200 pediatricians in different cities of SA-A. All fellows expressed high degree of satisfaction with their experience.

## Conclusion

This project developed an ongoing, successful educational strategy that may be used as a model for training in PR in other regions of the developing world. Evaluation of the impact of the program on care delivery will follow in the next few years.

## Disclosure of interest

None declared.

